# A case report of dangerous pelvic infundibulum ligament in the second trimester of pregnancy with massive hemorrhage

**DOI:** 10.1097/MD.0000000000035230

**Published:** 2023-09-22

**Authors:** Jiahui Dai, Jin Chen, Xiaohan Xu, Ni Gao, Yunfei Wang

**Affiliations:** a Clinical Medical College, Jining Medical University, Jining, Shandong, China; b Program for Scientific Research Innovation Team in Precision Medicine of Gynecologic Oncology, Affiliated Hospital of Jining Medical University, Jining, Shandong China.

**Keywords:** abdominal pregnancy, infundibulum ligament, massive bleeding, second trimester

## Abstract

**Rationale:**

Abdominal pregnancy is a rare ectopic pregnancy and its diagnosis and treatment are more challenging than those of other ectopic pregnancies. Because of a variable pregnancy site, abdominal pregnancy is associated with an increased risk of fatal abdominal hemorrhage, and consequently, an increased risk of maternal death compared with intrauterine pregnancy.

**Diagnoses:**

Pelvic infundibulum ligament pregnancy complicated with massive hemorrhage.

**Patient concerns:**

42-year-old pregnant woman who did not undergo an obstetric examination during the first trimester presented with sudden abdominal pain during the second trimester. Abdominal pregnancy was confirmed after emergency treatment, causing difficulty in the comprehensive preoperative evaluation.

Interventions: In order to save the patient life, we actively carried out surgical treatment.

**Outcomes:**

The patient recovered well after the operation and was discharged on the 11th postoperative day. Blood β-human chorionic gonadotropin (β-hCG) levels and routine blood test results were normal 1 month after the surgery, and the patient had recovered.

**Lessons:**

Several challenges are encountered in the diagnosis of abdominal pregnancy with regard to insufficient economic, cultural, and medical resources. In case of ectopic pregnancies, surgery should be the first choice of treatment, and preparations of blood transfusion are essential to combat the risk of rapid hemorrhagic shock caused by placenta implantation in the infundibulum ligament of the pelvis. The operation must be performed by experienced obstetricians and gynecologists.

## 1. Introduction

Abdominal pregnancy refers to a serosal pregnancy occurring in the abdominal cavity, but it excludes tubal, ovarian, or broad ligament pregnancies. Abdominal pregnancy is an extremely rare ectopic pregnancy.^[1]^ Most intra-abdominal pregnancies are believed to be caused by ruptured fallopian tubes or reimplantation following miscarriage, accounting for approximately 0.6% to 4.0% of all ectopic pregnancies. Abdominal pregnancy causes severe complications such as abdominal hemorrhage and abdominal cavity organ rupture, which account for its mortality rate 7.7 times that of tubal pregnancy and 89.9 times that of intrauterine pregnancy.^[2–4]^ Here, we report a case of ovarian ligament propria implantation at 13 weeks of gestation, along with ovarian artery rupture and massive hemorrhage.

## 2. Case presentation

**Figure 1.**
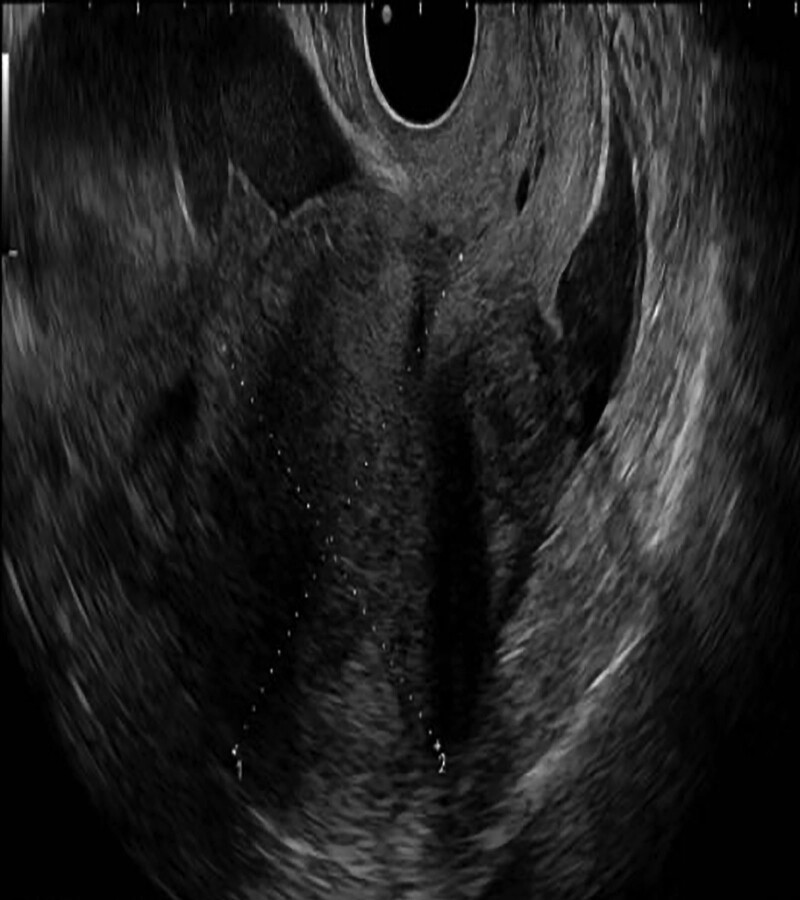
Ultrasound shows a fetal echo above the uterus in the abdominal cavity.

**Figure 2.**
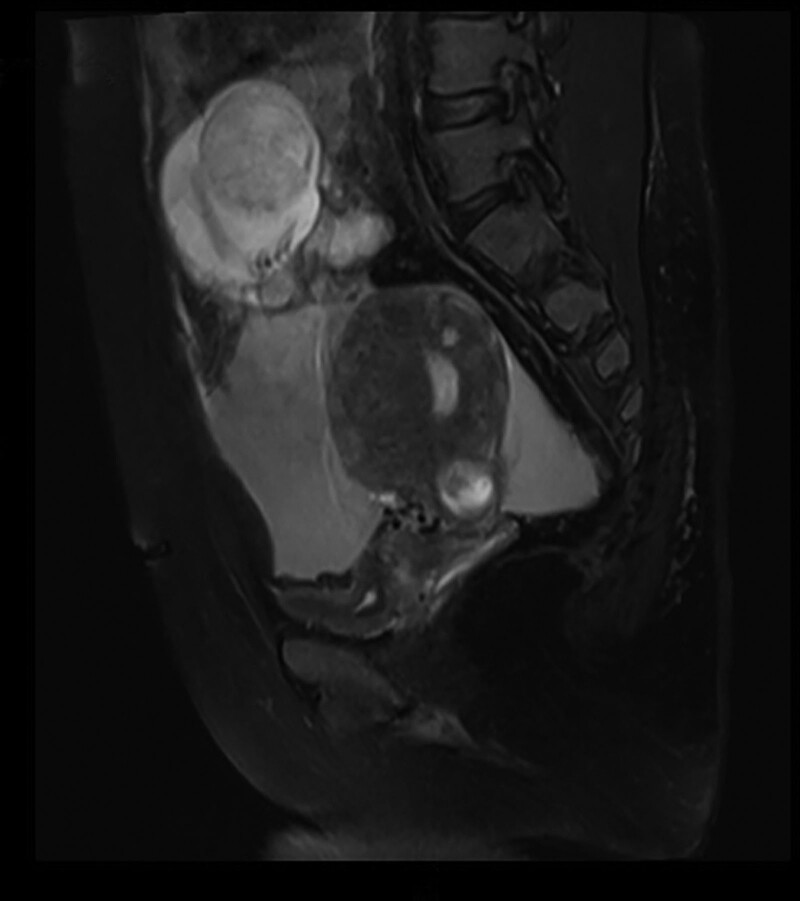
MRI shows an abdominal pregnancy with the fetus above the uterus. MRI = magnetic resonance imaging.

The patient was a 42-year-old woman having a history of 2 pregnancies, with 1 normal delivery and 1 full-term cesarean section. She complaint of “13 + 2 weeks of menopause and 1 day of abdominal pain,” following which she was admitted to the hospital on June 17, 2022. Her last menstrual period was March 16, 2022. After >30 days of menopause, the urine pregnancy self-test was positive, and she had not undergone any prenatal examinations. One day before admission, no obvious causes of severe pain in the upper abdomen were noted. She experienced nausea and vomiting, and the pain later shifted to the lower abdomen. Anal swelling or vaginal bleeding was absent, which could be resolved after a period of rest. On the day of admission, the abovementioned symptoms reappeared; however, this time the lower abdominal pain persisted and was more intense than before; hence, she visited the emergency department of our hospital. Emergency color ultrasound revealed that the uterus was enlarged, and the uterine cavity line was separated by approximately 1.0 cm. A fetal echo was observed above with a crest-rump diameter of approximately 9.9 cm, biparietal diameter of approximately 4.3 cm, femur length of approximately 2.0 cm, good fetal heart rate fluctuation, and a maximum depth of amniotic fluid of approximately 4.9 cm (Fig. 1). The laboratory parameters were examined, and the values are as follows: β-hCG level: 132364.00 mIU/mL; blood routine: WBC = 17.09 × 10^9^/L, RBC = 3.41 × 10^12^/L, Hb = 106 g/L, and neutrophils = 93.00%; coagulation: plasma fibrinogen = 4.7 g/L and D-dimer = 5.73 mg/L. The remaining parameters showed no obvious abnormalities. Magnetic resonance imaging (MRI) confirmed abdominal pregnancy, and the fetus was in the upper right of the uterus (Fig. 2).

**Figure 3.**
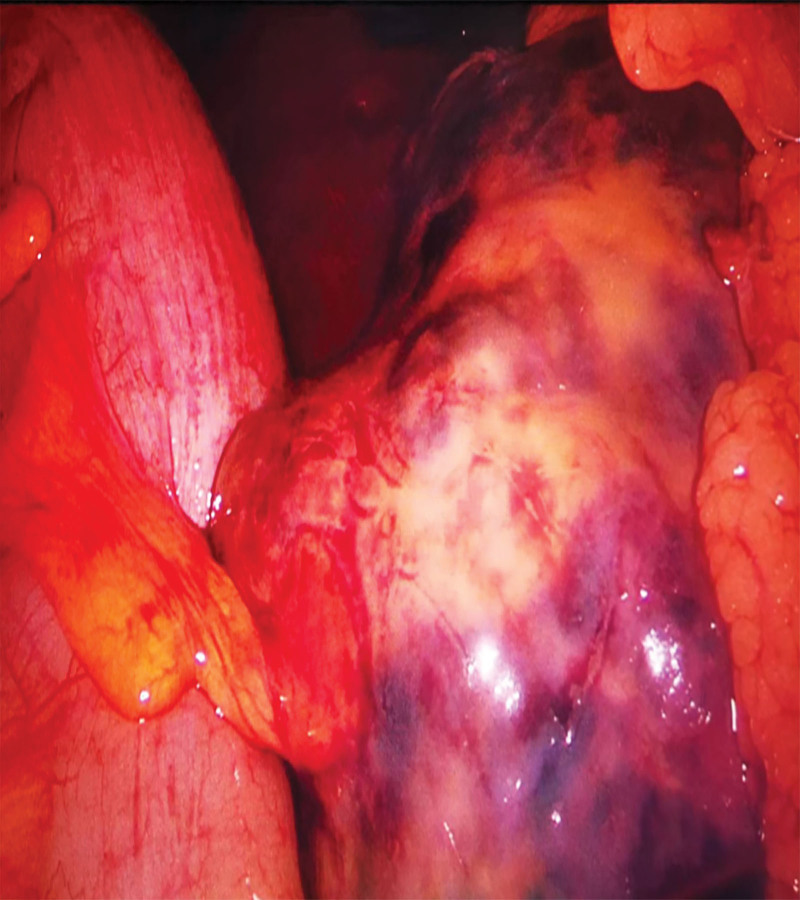
A purple-blue mass with a diameter of about 15 cm on the right side of the pelvic cavity is closely related to the surrounding tissues.

Because the patient was experiencing persistent abdominal pain, emergency laparoscopic exploratory surgery had to be performed on June 18, 2022 after appropriate preoperative preparations. A large amount of previous hemorrhage and blood clots were observed in the pelvis during the operation, and a purple-blue mass with a diameter of approximately 15 cm was detected on the right side of the pelvic abdomen, which was densely adhered to the intestine and omentum with an unclear boundary (Fig. 3). The appearances of the left fallopian tube and ovary were unremarkable.

**Figure 4.**
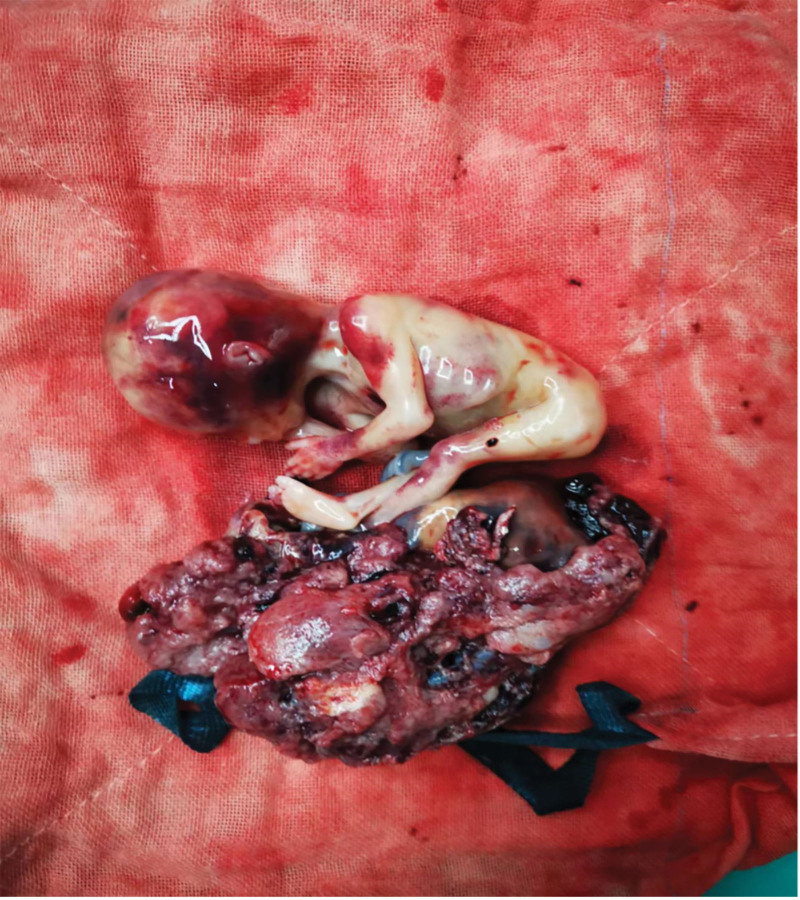
Fetus and its subsidiary tissues.

The pelvic and abdominal mass were carefully separated from the surrounding tissue adhesion layer by layer. Because of compression release, a large amount of blood suddenly gushed out and instantly filled the pelvic and abdominal cavities. The patient blood pressure sharply dropped to 52/23 mm Hg. The anesthesiologist quickly expanded the volume, increased venous access, and transfused red blood cells, platelets, and plasma. On creating an upper longitudinal incision of approximately 10 cm in length, laparotomy revealed a large amount of blood that poured out from the abdominal cavity. Gauze pads were placed on the pelvic abdominal cavity to stop bleeding; however, a large amount of blood continued to gush out of the abdominal cavity. The incision was immediately extended to the umbilicus, with a total length of approximately 16 cm and administration was continued. A total of 44 gauze pads were stuffed in the pelvic cavity to stop bleeding. When the blood pressure returned to 107/51 mm Hg, the stuffed gauze pads were removed and the pelvic abdominal hemorrhage and blood clots were cleaned. The pelvic abdominal mass pedicle was observed in the right pelvis. At the infundibulum ligament, a rupture with a diameter of approximately 1 cm was observed on the surface along with active bleeding. Hemostasis was released using forceps, and the adhered pelvic abdominal mass, omentum, and intestinal tube were carefully separated. The fetus and placenta tissue were observed, and the fetus was completely removed and disassembled down the placental tissue (Fig. 4). However, the pregnancy tissue was still found to be partially implanted in the right ovarian artery and vein, with the other part of it being implanted in the right fallopian tube and ovary. The anatomical structure of the right adnexa was abnormal, which was thus removed after consultation with the patient family members.

**Figure 5.**
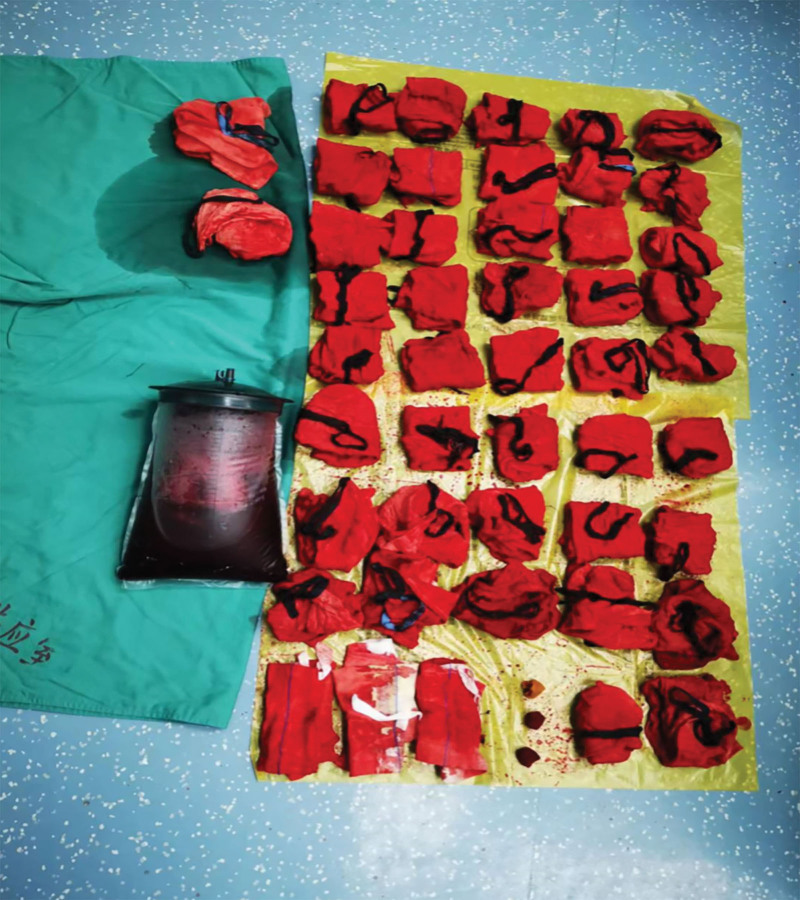
The blood loss during the operation was about 800 mL.

**Figure 6.**
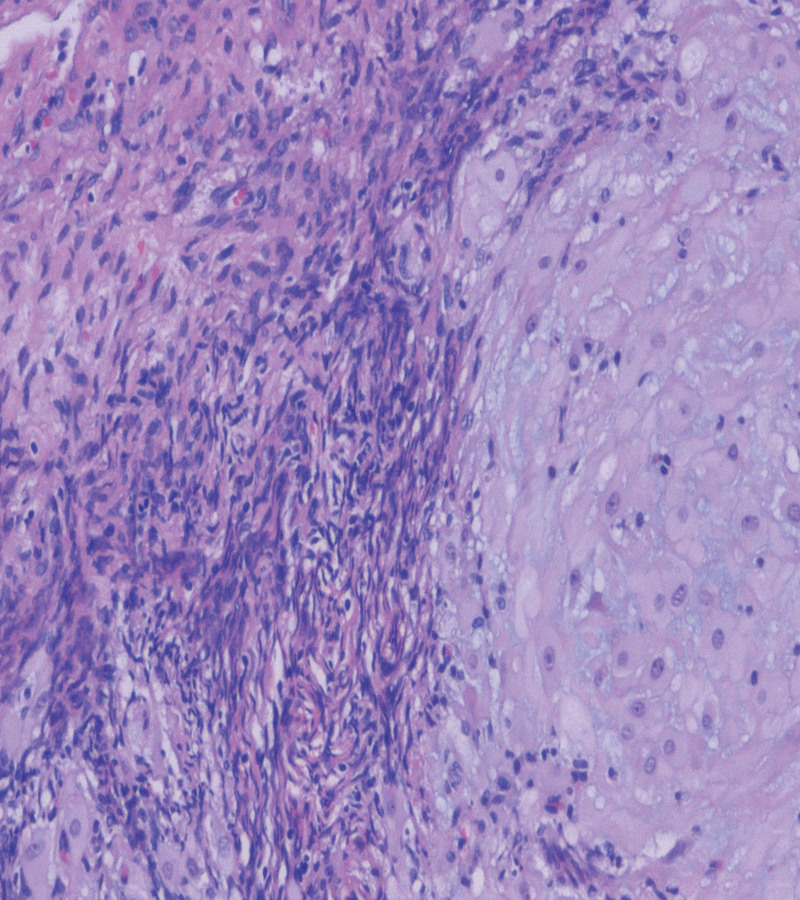
Pathological results revealed the following: (abdominal cavity) 1 placenta with 1 umbilical cord; degenerated placental thread hair and decidua tissue with hemorrhage and necrosis; slight neutrophil infiltration under part of the placenta; local placental villi close to the serosal surface of the fallopian tube; no changes in the umbilical cord and fetal tissue: (Appendix right) the ovarian tissue was sent for inspection. Decidua nodules and villi were observed on the surface, decidua, and smooth muscle tissue and were accompanied by hemorrhage and necrosis; villous tissue was observed in the local smooth muscle tissue.

During the operation, 3400 mL of pelvic hemorrhage-associated blood and clots were aspirated and approximately 800 mL of blood loss occurred (Fig. 5). Subsequently, the patient was infused with 14 U of leukocyte-free suspension red blood cells, 12 U of cryoprecipitate, 1000 mL of plasma, and a therapeutic dose of 1 unit of platelets The patient blood pressure after the operation was 120/70 mm Hg. Pathological results revealed the following: (abdominal cavity) 1 placenta with 1 umbilical cord; degenerated placental thread hair and decidua tissue with hemorrhage and necrosis; slight neutrophil infiltration under part of the placenta; local placental villi close to the serosal surface of the fallopian tube; no changes in the umbilical cord and fetal tissue: (Appendix right) the ovarian tissue was sent for inspection. Decidua nodules and villi were observed on the surface, decidua, and smooth muscle tissue and were accompanied by hemorrhage and necrosis; villous tissue was observed in the local smooth muscle tissue (Fig. 6).

The patient recovered well after the operation and was discharged on the 11^th^ postoperative day. Blood β-HCG levels and routine blood test results were normal 1 month after the surgery, and the patient had recovered.

## 3. Discussion

In abdominal pregnancy, the gestational sac is planted in the abdominal cavity outside the uterus, fallopian tubes, and ovaries. It is an extremely rare type of ectopic pregnancy, with an incidence rate of 1:10000 to 1:30000, accounting for 0.6% of all ectopic pregnancies.^[2,5]^ Most abdominal pregnancies originate from ruptured tubal pregnancies, and in some cases, the implantation occurs directly on the uterine serosa, omentum, intestine, and mesentery, thereby increasing the risk of fatal intra-abdominal hemorrhage.^[6]^ Reportedly, the maternal mortality rate of abdominal pregnancy is approximately 7.7 times that of tubal pregnancy.^[3]^ As observed in the present case, in abdominal pregnancy, the implantation site is variable and the ovarian arteries and veins are eroded, necessitating operation under urgent circumstances, and bleeding is severe, which is extremely challenging to control and is dangerous. Therefore, early diagnosis and appropriate treatment are crucial to reduce the mortality rate associated with abdominal pregnancy.

Abdominal pregnancy is divided into primary and secondary abdominal pregnancies. Primary intra-abdominal pregnancy is relatively rare and is the direct implantation of fertilized eggs into the abdominal cavity. This should be diagnosed according to the diagnostic criteria proposed by Studdiford^[1]^: normal fallopian tubes and ovaries; no evidence of uterine-peritoneal fistula; pregnancy is only related to the peritoneal surface; and no evidence of secondary implantation after primary tubal implantation. Secondary intra-abdominal pregnancy is more common and is often the result of peritoneal implantation following rupture of the fallopian tube, ovary, or intrauterine pregnancy.^[5,7]^ In the present case, the patient uterus was intact during the operation, the left adnexa appeared normal, and the normal anatomical structure of the right adnexa was unclear. The postoperative pathology showed that the placental fetal surface had the umbrella end of the fallopian tube, which was considered to happen after the fallopian tube rupture. The fetus was free from the abdominal cavity, and then the placenta implanted into the ovary, ovarian arteriovenous, pelvic infundibulum ligament, and omentum to form a new blood supply system, followed by the onset of abdominal pregnancy.

The clinical manifestations of abdominal pregnancy are not specific and can be easily confused with those of general ectopic pregnancy such as tubal ectopic pregnancy. Early abdominal pregnancy is difficult to diagnose and is often misdiagnosed as tubal pregnancy. Ultrasound examination is the preferred diagnostic tool, which is effective during mid-to-late abdominal pregnancy. The examination shows an empty uterine cavity and the fetus is observed to be separated from the uterus. However, distinguishing early abdominal pregnancy from other ectopic pregnancies using ultrasonography is challenging. MRI not only plays a significant role in diagnosing mid-to-late abdominal pregnancy but is also more accurate in evaluating organ involvement because it can clearly show soft tissues. Therefore, preoperative MRI is recommended for patients. MRI can also be useful in diagnosing early abdominal pregnancy when the pregnancy location is unclear. In the present case, because the patient did not undergo an early pregnancy checkup, when the symptoms appeared, abdominal ultrasound was used to diagnose abdominal pregnancy, and abdominal MRI examination was subsequently performed for confirmation. Simultaneously, MRI was used to accurately observe the placenta, fetus, and abdominal viscera. However, it is still difficult to particularly classify the case as primary or secondary abdominal pregnancy. This can only be speculated via intraoperative and postoperative pathology. Based on the observations, we assume that the current case is secondary abdominal pregnancy.

The risk factors for a secondary intra-abdominal pregnancy are the same as those for an ectopic pregnancy. Secondary intra-abdominal pregnancy is extremely dangerous; the expected treatment can cause sudden fatal intra-abdominal hemorrhage and the prognosis of the fetus is often poor. It is recommended to perform active surgical treatment with sufficient preoperative preparation. As observed in this patient during the operation, the fetus, placenta, right appendage, pelvic infundibulum ligament, omentum, and intestinal tube were densely adhered to form an encased compression. Saving lives of patients wins them precious time. Therefore, immediately terminating the pregnancy after a confirmed diagnosis is highly recommended, and the most effective treatment option is the surgical removal of the fetus by laparoscopy or exploratory laparotomy. However, when the fetus is viable for birth, intervention can be delayed in favor of a newborn delivered via cesarean section.^[8–10]^

## 4. Conclusion

Abdominal pregnancy is an extremely rare ectopic pregnancy. Modern medicine has greatly developed and most abdominal pregnancy cases can be diagnosed and terminated during early pregnancy; however, certain challenges remain for remote regions with insufficient medical resources.

Surgery is the first choice of treatment for abdominal pregnancy with pelvic infundibulum ligament implantation. The anatomical relationship between the placenta and surrounding tissues should be carefully explored, and massive bleeding and organ damage should be avoided to the maximum possible extent. All necessary preparations must be made before the operation, such as actively preparing blood for transfusion and requirements for stopping blood clots in case of abdominal hemorrhage. Patient family members should be actively consulted, and appendages on the affected side should be removed if necessary. Overall, this case report guides the diagnosis and treatment of abdominal pregnancy.

## Acknowledgments

The author thanks our hospital colleagues for their support and the patient dedication.

## Author contributions

**Formal analysis:** Jiahui Dai.

**Investigation:** Jiahui Dai.

**Methodology:** Jiahui Dai.

**Visualization:** Jin Chen, Xiaohan Xu.

**Writing – original draft:** Jiahui Dai, Jin Chen, Ni Gao.

**Writing – review & editing:** Jiahui Dai, Yunfei Wang.
